# Complete Genome Sequence of *Escherichia coli* BLR(DE3), a *recA*-Deficient Derivative of *E. coli* BL21(DE3)

**DOI:** 10.1128/genomeA.00441-17

**Published:** 2017-06-01

**Authors:** Philippe Goffin, Philippe Dehottay

**Affiliations:** a GSK Vaccines, Rixensart, Belgium; b Laboratoire de Génétique et Physiologie Bactérienne, IBMM, Faculté des Sciences, Université Libre de Bruxelles (ULB), Gosselies, Belgium

## Abstract

*Escherichia coli* BLR(DE3) is a commercially available *recA*-deficient derivative of BL21(DE3), one of the most widely used strains for recombinant protein expression. Here, we present the full-genome sequence of BLR(DE3) and highlight additional differences with its parent strain BL21(DE3) which were previously unreported but may affect its physiology.

## GENOME ANNOUNCEMENT

*Escherichia coli* is widely used for the production of recombinant polypeptides, including therapeutic proteins (1). The most popular system for protein overexpression in *E. coli* is the isopropyl-β-d-thiogalactopyranoside (IPTG)-inducible T7 RNA polymerase cascade system, in which expression of the gene of interest is controlled by a T7 RNA polymerase promoter (e.g., on a pET vector) in a strain carrying the T7 RNA polymerase gene under control of an IPTG-inducible promoter (e.g., λDE3 lysogenic strains) (2). The genome sequence of the widely used *E. coli* B strain BL21(DE3) (2) is available (3). BLR(DE3) is a *recA*-deficient derivative of BL21(DE3) that helps stabilize plasmids containing repetitive sequences (4). According to Novagen, BLR(DE3) has the same genotype, except for a mutation in *recA* [Δ(*srl*-*recA*)306::Tn*10*], which was initially obtained in an *E. coli* K-12 background (5) and later transferred to BL21 (4, 6).

We sequenced the BLR(DE3) strain obtained from Novagen in order to verify whether additional differences are present in BLR(DE3) compared to BL21(DE3). We performed *de novo* hybrid assembly from PacBio sequencing (102,708 reads, with a mean length of 5,173 bp) and Illumina HiSeq 2000 sequencing (approx. 4,500,000 high-quality paired-end 100-bp reads) for closing gaps and correcting PacBio sequencing errors. A total of 4,306 coding sequences (CDSs), 230 pseudogenes, 85 tRNA genes, 22 rRNA genes (8 operons), and 64 noncoding RNA genes were annotated.

A comparison with BL21(DE3) indicates larger differences than expected, with two main divergent regions. First, the DE3 prophage contains three large deletions of 4.9 kb (*cro*-*Rz*), 1.8 kb (*A-B*), and 11.1 kb (*Fi*-*J*), suggesting the prophage is nonfunctional. As expected, the second divergent region is the *recA* locus. A 6.9-kb region from *recA* to *srlR* is replaced with Tn*10* in BLR(DE3). Besides *recA* and the *srl* operon, BLR(DE3) thus also lacks *pncC* (encoding nicotinamide mononucleotide deamidase, a key enzyme of the pyridine nucleotide cycle [7]) and *mtlB* (encoding a lytic murein transglycosylase). Downstream of Tn*10*, a large region (approx. 75 kb; *srlR*-*queE*) diverges significantly from BL21(DE3), with 577 nucleotide substitutions (1 to 3 bp), short insertions/deletions (1 bp), and the replacement of an IS*186* element with a full clustered regularly interspaced short palindromic repeat (CRISPR) locus, as found in *E. coli* MG1655. We infer that the entire ~85-kb region from *recA* to *queE* was transferred from the original K-12 background bearing the Δ(*srl*-*recA*)306::Tn*10* mutation. This region contains several genes involved in metabolism and other key processes, such as DNA mismatch repair (*mutS*) or stress response (*rpoS*). Importantly, *rpoS* carries a nonsense mutation at codon 33, which has been shown to reduce σ^S^ activity (8). Outside the DE3 and *recA* loci, BLR(DE3) and BL21(DE3) contain fewer than 20 differences. Of note, these include a previously identified mutation in *ilvA*, a gene responsible for isoleucine auxotrophy (9).

Globally, the genome sequence of BLR(DE3) indicates that it cannot be merely considered a *recA*-deficient BL21(DE3) strain. The newly identified differences might contribute to a differential physiology of BLR(DE3), besides its reported impaired homologous recombination.

### Accession number(s).

The complete genome sequence of *Escherichia coli* BLR(DE3) has been deposited at GenBank under accession number CP020368.
